# Machine Learning‐Driven Noninvasive Prediction of Liver Fibrosis in Unclassified Liver Disease

**DOI:** 10.1002/mco2.70990

**Published:** 2026-10-04

**Authors:** Ye Yang, Yirui Hu, Ting Wei, Yue Chen, Wenkang Gao, Yupin Tan, Yuanqing Zhu, Yonggang Yuan, Zhuang Cao, Yunfei Zhao, Zexuan Li, Jiake Che, Yue Yang, Xinyang Wang, Shengqi Yan, Weiyan Huang, Shuyan Wang, Fuyu Yang, Lijia Yin, Xiaotong Du, Jiahao Liu, Rong Lin, Ling Yang, Huikuan Chu

**Affiliations:** ^1^ Division of Gastroenterology Union Hospital, Tongji Medical College, Huazhong University of Science and Technology Wuhan Hubei China; ^2^ Department of Critical Care Medicine Zhongnan Hospital of Wuhan University Wuhan China; ^3^ Clinical Research Center of Hubei Critical Care Medicine Wuhan China

**Keywords:** CatBoost, chronic liver disease, liver fibrosis, machine learning, noninvasive staging

## Abstract

Chronic liver diseases (CLDs) frequently progress silently to advanced fibrosis or cirrhosis, yet existing predictive models have largely been developed for patients with clearly defined etiologies. Here, we asked whether a noninvasive machine‐learning model based on routine clinical variables and liver stiffness measurement (LSM) could accurately identify significant liver fibrosis in patients with CLD of uncertain etiology, and whether such a model would add value beyond conventional fibrosis scores for clinical decision‐making. We retrospectively analyzed 503 patients who underwent liver biopsy at Union Hospital, Tongji Medical College, Huazhong University of Science and Technology. Twelve machine‐learning algorithms were trained to predict significant hepatic fibrosis, and model interpretability was assessed using SHapley Additive exPlanations. The best‐performing model was then compared with four established noninvasive tools: FIB‐4, GPR, Forns Index, and LSM alone. Among all candidate models, Categorical Boosting delivered the most favorable overall diagnostic performance, achieving the highest discriminative accuracy (AUC, 0.827; 95% CI, 0.754–0.900) and outperforming all conventional comparators across key classification metrics. We therefore establish an interpretable and clinically applicable panel integrating serological markers, age, LSM for noninvasive fibrosis assessment in patients with undefined or clinically ambiguous liver disease, with potential to reduce unnecessary biopsy and enable earlier intervention.

## Graphical abstract

Using a biopsy‐confirmed cohort of 503 patients with chronic liver disease of uncertain or clinically ambiguous etiology, we developed an interpretable machine‐learning framework for the noninvasive identification of significant liver fibrosis, defined as Scheuer stage S≥2. Seven routinely available variables—age, PLT, AST, GGT, ALB, LSM, and FIB‐4—were integrated into twelve candidate algorithms. CatBoost was selected as the final diagnostic prediction model, achieving an AUROC of 0.827 and PR‐AUC of 0.804 in the independent test cohort, outperforming FIB‐4, GPR, Forns Index, and LSM. This model may support early fibrosis risk stratification and biopsy‐sparing clinical decision‐making.

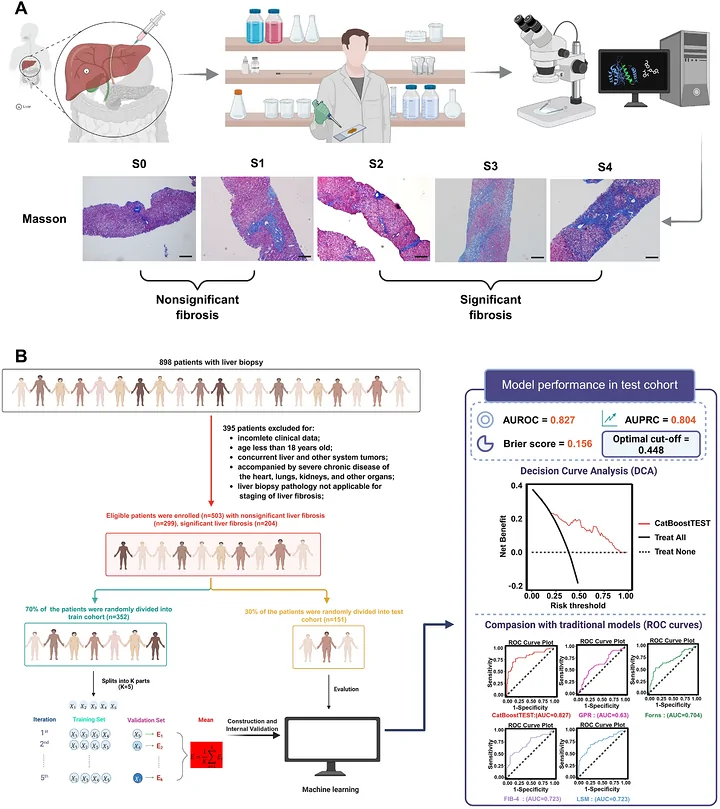

## 1 Introduction

Liver fibrosis represents a critical pathological hallmark of chronic liver diseases (CLDs), underpinning the progression toward liver cirrhosis and hepatocellular carcinoma, characterized by hepatic stellate cell activation, excessive extracellular matrix deposition, and subsequent disruption of hepatic architecture [1]. Globally, the prevalence of CLDs, such as metabolic‐associated steatotic liver disease, viral hepatitis, and alcohol‐induced liver disease, continues to increase significantly, with recent estimates indicating that more than 1.5 billion individuals are affected by varying degrees of chronic hepatic disorders, leading to approximately 2 million liver disease‐related deaths annually, primarily driven by advanced fibrosis and its complications [2]. Notably, CLDs frequently progress without overt symptoms, resulting in many patients being hospitalized with liver dysfunction before a definitive etiological classification can be established. Moreover, initiating treatment during the early stages of hepatic fibrosis can effectively suppress fibrogenesis and, to a certain degree, promote its reversal. This therapeutic approach is closely associated with improved long‐term clinical outcomes [3]. It is therefore crucial to recognize individuals with chronic liver conditions who are at elevated risk of disease progression and who could potentially benefit from timely medical intervention [4].

Traditionally, liver biopsy remains the gold standard for fibrosis staging; however, it has significant drawbacks including invasiveness, associated complications, and sampling variability, thereby limiting its utility for population‐wide screening and longitudinal patient management [5]. To overcome these limitations, various noninvasive diagnostic methods, including transient elastography and serum‐based biomarkers (e.g., APRI, FIB‐4, and FORN scores), have been widely implemented in clinical settings [6, 7, 8, 9]. Nonetheless, these models have yielded inconsistent and sometimes contradictory results across different study populations. That is, these noninvasive methods still exhibit notable limitations in terms of diagnostic accuracy and reproducibility, which restrict their utility in precise clinical decision‐making [10, 11, 12]. Integrating additional indicators is needed to enhance their clinical discriminative performance.

In recent years, artificial intelligence, particularly machine learning (ML) algorithms, has emerged as a powerful tool for extracting intricate patterns from clinical data, significantly enhancing diagnostic and prognostic capabilities in CLDs [13]. For instance, in patients with chronic hepatitis B complicated by hepatic steatosis, the RF‐AF and RF‐C models exhibit superior diagnostic performance for the detection of advanced fibrosis and cirrhosis, characterized by improved accuracy and a notably lower rate of missed diagnoses than conventional noninvasive tests [14]. Moreover, ensemble learning algorithms, notably Categorical Boosting (CatBoost), eXtreme Gradient Boosting (XGBoost), and Light Gradient Boosting Machine (LightGBM), have demonstrated robust predictive accuracy and stability across various clinical scenarios [15, 16, 17]. While ML‐based noninvasive assessment tools have been applied in certain defined liver disease subtypes, no ML‐based fibrosis staging models have been developed specifically for etiologically ambiguous patients. Their diagnosis still relies primarily on liver biopsy.

In this study, we aimed to address a clinically relevant question: whether a noninvasive ML model integrating routinely available clinical variables, liver stiffness measurement (LSM) and FIB‐4 can accurately identify significant liver fibrosis, defined as Scheuer stage S≥2, in patients with CLD whose etiology has not yet been definitively established or remains clinically ambiguous. We further sought to determine which of the 12 candidate ML algorithms achieved the best predictive performance in this setting, and whether the optimal model could outperform conventional noninvasive fibrosis assessment tools, including the GPR, Forns Index, LSM alone, and FIB‐4 alone. To address these questions, we developed and internally validated an interpretable ML‐based fibrosis prediction model using a biopsy‐confirmed cohort, with the goal of supporting earlier risk stratification and treatment decision‐making in this clinically challenging population.

## 2 Results

### 2.1 Patient Enrollment and Baseline Characteristics of the Study Cohort

This retrospective study initially reviewed 898 patients who underwent percutaneous liver biopsy at our institution (Figure 1A,B). After rigorously defined inclusion and exclusion criteria were applied, 503 eligible patients were included—345 women and 158 men, aged 18–77 years, with a median age of 49 years (Table 1). The etiological distribution of the enrolled cohort was as follows: 23 patients with viral liver disease, 56 with alcoholic liver disease, 63 with nonalcoholic fatty liver disease (NAFLD), 294 with autoimmune liver disease, 47 with drug‐induced liver injury, 18 with cholestatic liver disease, and one with a hereditary metabolic liver disorder. Fibrosis staging was performed histologically using the Scheuer score. Accordingly, 299 patients were categorized as having nonsignificant fibrosis (S0/S1) and 204 patients were categorized as having significant fibrosis (S≥2) (Figure 1A).

**FIGURE 1:**
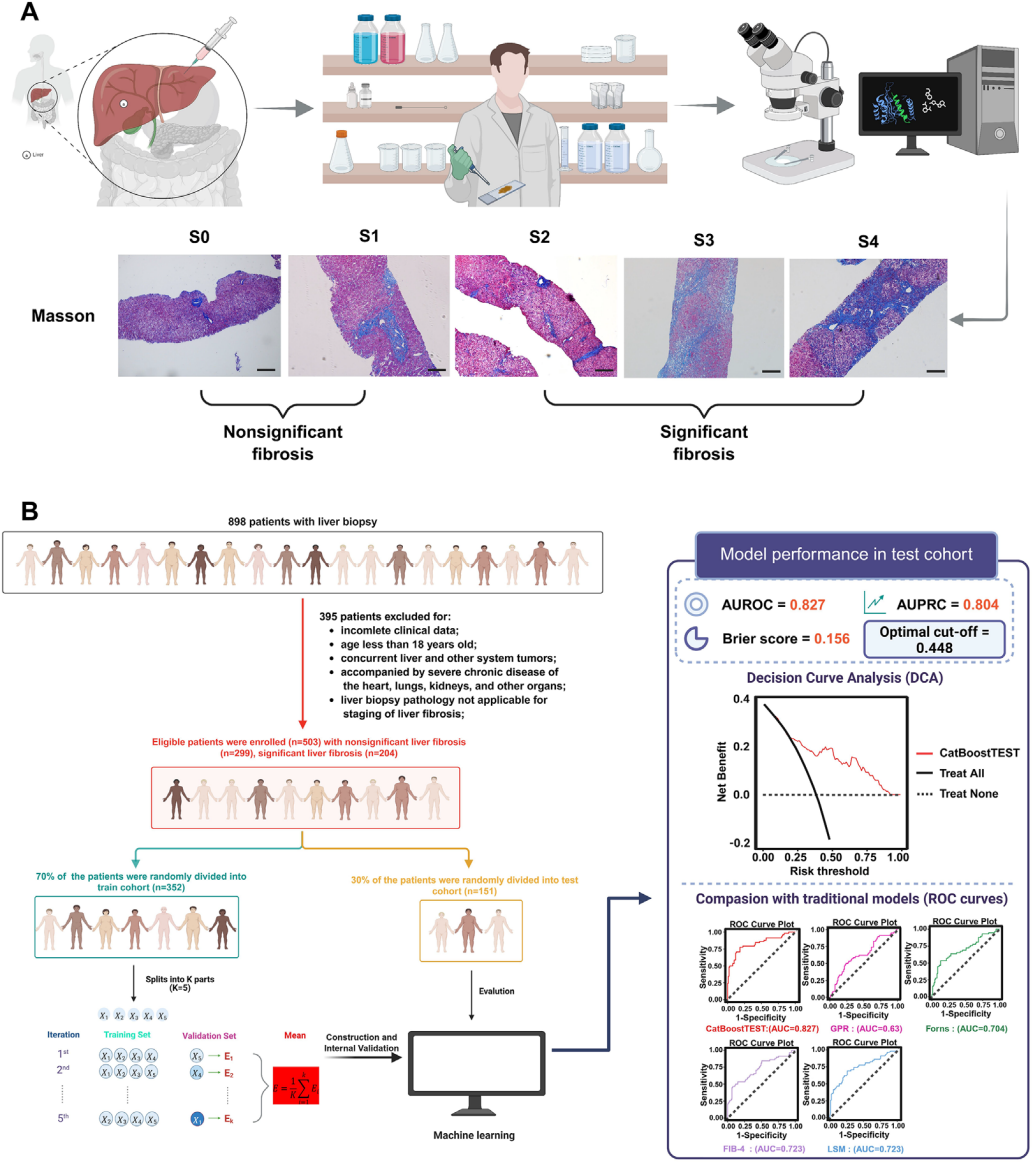
Study design and participant selection workflow. The study flowchart was created with BioRender.com. (A) Histological classification of liver fibrosis stages based on percutaneous liver biopsy among eligible participants. Scale bars indicate 500 µm. (B) Sequential framework of model development, including data preprocessing, training, internal validation, and deployment.

**TABLE 1:** Baseline characteristics of patients in the training and test cohorts.

Variables	Overall *n* = 503	Training set *n* = 352	Test set *n* = 151	*p* Value
Age (year)	49 (41–57)	50 (41–57)	49 (40.5–56)	0.354
BMI (kg/m^2^)	22.46 (20.535–24.69)	22.49 (20.57–24.84)	22.27 (20.405–24.415)	0.261
WBC (×10^9^/L)	5.11 (4.04–6.3)	5.14 (4.128–6.42)	4.89 (3.835–5.895)	0.087
RBC (×10^12^/L)	4.06 (3.76–4.45)	4.095 (3.8–4.47)	4.01 (3.71–4.37)	0.132
HB (×10^9^/L)	124 (115–134)	125 (117–134)	123 (112–135)	0.122
Hct (%)	37.3 (34.8–40.25)	37.6 (35.1–40.4)	36.7 (33.65–40)	0.049
N (×10^9^/L)	2.7 (1.91–3.585)	2.765 (2–3.52)	2.63 (1.85–3.75)	0.512
LY (×10^9^/L)	1.64 (1.27–2.1)	1.645 (1.3–2.102)	1.63 (1.16–1.965)	0.175
PLT (×10^9^/L)	192.205 ± 68.988	195.344 ± 68.936	184.887 ± 68.781	0.120
PAR	4.821 ± 1.69	4.906 ± 1.751	4.621 ± 1.526	0.068
SII	311.938	315.054	304.179	0.393
	(201.594–439.858)	(206.501–450.837)	(186.592–416.66)	
NPR	0.01 (0.01–0.02)	0.01 (0.01–0.02)	0.01 (0.01–0.02)	0.352
PLR	114.81	113.525	116.04	0.925
	(86.55–148.025)	(87.105–147.257)	(82.97–149.955)	
NLR	1.67 (1.21–2.23)	1.645 (1.2–2.212)	1.72 (1.25–2.275)	0.670
TB (µmol/L)	15.6 (10.8–23.85)	15.7 (10.7–25)	14.8 (11.15–22.95)	0.472
DB (µmol/L)	5.6 (3.7–10.5)	5.6 (3.6–10.5)	5.6 (4.2–10.35)	0.628
DB/TB	0.39 (0.32–0.49)	0.38 (0.318–0.48)	0.41 (0.33–0.51)	0.072
ALT (U/L)	53 (28–101.5)	52.5 (28.75–97.75)	58 (28–107.5)	0.585
AST (U/L)	49 (29–84.5)	51 (29–83.25)	48 (31–88.5)	0.749
AST/ALT	0.916 (0.683–1.414)	0.935 (0.715–1.457)	0.891 (0.649–1.291)	0.140
GGT(U/L)	92 (42–231.5)	88.5 (42–237.25)	99 (43.5–206.5)	0.970
GGT/AST	1.74 (0.85–3.735)	1.71 (0.825–3.775)	1.81 (0.895–3.7)	0.865
ALP(U/L)	101 (71–178)	101 (70–176.75)	102 (74.5–178)	0.732
ALB (g/L)	40.2 (37–43.5)	40.2 (37.375–43.425)	40.3 (36.5–43.5)	0.776
GLB (g/L)	27.9 (24.3–31.8)	27.85 (24.275–31.8)	27.9 (25.05–31.7)	0.765
A/G	1.468 (1.225–1.676)	1.478 (1.223–1.704)	1.437 (1.228–1.646)	0.487
TC (mmol/L)	4.61 (3.86–5.245)	4.625 (3.87–5.285)	4.56 (3.83–5.17)	0.471
TG (mmol/L)	1.25 (0.94–1.78)	1.26 (0.97–1.75)	1.23 (0.885–1.915)	0.607
HDL (mmol/L)	1.29 (0.96–1.55)	1.29 (0.97–1.57)	1.29 (0.96–1.52)	0.288
LDL (mmol/L)	2.63 (2.105–3.18)	2.675 (2.138–3.17)	2.54 (2.05–3.23)	0.361
Cr (µmol/L)	53.6 (39.6–66.8)	55.05 (37–67.775)	53 (43.1–63)	0.576
BUN (mmol/L)	4.78 (3.865–8.16)	4.78 (3.86–10.01)	4.79 (3.89–6.47)	0.343
UA (µmol/L)	267 (208.35–343.85)	267.1 (205.4–345.475)	265.9 (213.85–342.85)	0.859
LDH (U/L)	190 (138.5–231)	191 (135.75–232)	187 (149–227)	0.881
Creatine kinase (U/L)	77 (49–109.815)	79.93 (52–110.24)	70 (41–108.56)	0.097
eGFR (mL/min/1.73 m^2^)	101.26 (70.64–111.61)	99.08 (59.698–111.74)	104.43 (87.075–111.32)	0.117
K (mmol/L)	4.04 (3.79–4.5)	4.05 (3.8–4.695)	4 (3.78–4.295)	0.307
Na (mmol/L)	139.6 (135–141.6)	139.5 (133.075–141.5)	140 (137.15–141.75)	0.244
Cl (mmol/L)	101.7 (97.5–103.7)	101.7 (96.15–103.6)	101.9 (98.45–104.15)	0.252
Ca (mmol/L)	2.2 (2.035–2.29)	2.19 (2.01–2.3)	2.2 (2.06–2.27)	0.788
Mg (mmol/L)	0.88 (0.82–0.96)	0.89 (0.82–0.97)	0.87 (0.82–0.95)	0.112
P (mmol/L)	1.12 (0.98–1.39)	1.12 (0.98–1.422)	1.12 (0.995–1.295)	0.903
CO_2_ (mmol/L)	23.7 (20.75–26)	23.5 (19.525–25.9)	24.1 (22.15–26.25)	0.050
D‐dimer (mg/L FEU)	0.22 (0.22–0.4)	0.22 (0.22–0.4)	0.22 (0.22–0.395)	0.920
PT (s)	12.8 (12.3–13.6)	12.8 (12.3–13.6)	12.9 (12.3–13.6)	0.682
INR	1.01 (0.94–1.09)	1.01 (0.93–1.09)	1.01 (0.95–1.105)	0.288
APTT (s)	36.8 (34.65–40.35)	36.75 (34.6–40.325)	36.9 (34.75–40.35)	0.713
FIB (g/L)	2.71 (2.285–3.25)	2.68 (2.26–3.243)	2.73 (2.33–3.325)	0.552
TT (s)	18.6 (17.4–19.7)	18.6 (17.4–19.725)	18.5 (17.3–19.4)	0.259
LSM (kPa)	9.5 (6.587–12.877)	9.5 (6.634–12.555)	9.55 (6.519–13.349)	0.397
FIB‐4	0.498 (0.289–0.889)	0.508 (0.282–0.884)	0.456 (0.312–0.88)	0.795
Gender (%)				0.665
Female	345 (68.59)	244 (69.32)	101 (66.89)	
Male	158 (31.41)	108 (30.68)	50 (33.11)	
DM (%)				0.365
No	452 (89.86)	313 (88.92)	139 (92.05)	
Yes	51 (10.14)	39 (11.08)	12 (7.95)	
HTN (%)				0.769
No	421 (83.70)	293 (83.24)	128 (84.77)	
Yes	82 (16.30)	59 (16.76)	23 (15.23)	
CHD (%)				1.000
No	466 (92.64)	326 (92.61)	140 (92.72)	
Yes	37 (7.36)	26 (7.39)	11 (7.28)	
HLP (%)				0.022
No	303 (60.24)	200 (56.82)	103 (68.21)	
Yes				
	200 (39.76)	152 (43.18)	48 (31.79)	
Viral liver disease				0.590
No	12 (52.17)	9 (47.37)	3 (75.00)	
Yes	11 (47.83)	10 (52.63)	1 (25.00)	
Alcoholic liver disease				1.000
No	8 (13.79)	6 (15.00)	2 (11.11)	
Yes	50 (86.21)	34 85.00)	16 (88.89)	
NAFLD				1.000
No	55 (87.30)	40 (86.96)	15 (88.24)	
Yes	8 (12.70)	6 (13.04)	2 (11.76)	
Autoimmune liver disease				0.675
No	176 (59.86)	120 (58.82)	56 (62.22)	
Yes	118 (40.14)	84 (41.18)	34 (37.78)	
Drug‐induced liver injury				1.000
No	33 (70.21)	22 (68.75)	11 (73.33)	
Yes	14 (29.79)	10 (31.25)	4 (26.67)	
Cholestatic liver disease				1.000
No	14 (77.78)	9 (81.82)	5 (71.43)	
Yes	4 (22.22)	2 (18.18)	2 (28.57)	
Hereditary metabolic liver disorder				
No	1 (100.00)	0 (0)	1 (100.00)	
Yes	0 (0)	0 (0)	0 (0)	
Significant fibrosis (%)				0.587
No (S0/1)	299 (59.44)	206 (58.52)	93 (61.59)	
Yes (S≥2)	204 (40.56)	146 (41.48)	58 (38.41)	

Data are *n* (%) or percentage (95%).

Abbreviations: BMI, body mass index; WBC, white blood cell; RBC, red blood cell; HB, hemoglobin; Hct, hematocrit; N, neutrophil; LY, lymphocyte; PLT, platelet; PAR, platelet–albumin ratio; SII, systemic inflammation index; NPR, neutrophil‐to‐platelet ratio; PLR, platelet‐to‐lymphocyte Ratio; NLR, neutrophil‐to‐lymphocyte ratio; TB, total bilirubin; DB, direct bilirubin; DB/TB, direct Bilirubin/Total Bilirubin; ALT, alanine aminotransferase; AST, aspartate aminotransferase; AST/ALT, aspartate aminotransferase/alanine aminotransferase; GGT, γ‐glutamyl transferase; GGT/AST, γ‐glutamyl transferase/aspartate aminotransferase; ALP, alkaline phosphatase; ALB, albumin; GLB, globulin; A/G, albumin/globulin; TC, total cholesterol; TG, triglyceride; HDL, high‐density lipoprotein; LDL, low‐density lipoprotein; Cr, creatinine; BUN, blood urea nitrogen; UA, uric acid; LDH, lactate dehydrogenase; eGFR, estimated glomerular filtration rate; PT, prothrombin time; INR, international normalized ratio; APTT, activated partial thromboplastin time; FIB, fibrinogen; TT, thrombin time; LSM, liver stiffness measurement; FIB‐4, fibrosis‐4 index; DM, diabetes mellitus; HTN, hypertension; CHD, coronary heart disease; HLP, hyperlipidemia; NAFLD, nonalcoholic fatty liver disease.

Using stratified random sampling method, the cohort was divided into a training set (*n* = 352) and a test set (*n* = 151) at a 7:3 ratio using SPSS software (Figure 1B). In the training set, 206 patients (58.52%) had nonsignificant fibrosis and 146 (41.48%) had significant fibrosis. In the test set, 93 patients (61.59%) had nonsignificant fibrosis and 58 (38.41%) has significant fibrosis. With the exception of Hct, no statistically significant differences in demographic characteristics or biochemical indices were observed between the training and test cohorts (Table 1).

Baseline laboratory comparisons within the training cohort revealed statistically significant differences in age, WBC, RBC, HB, Hct, LY, PLT, PAR, NPR, NLR, TB, DB, DB/TB, AST, AST/ALT, GGT, ALP, ALB, GLB, A/G, TC, LDL, P, D‐dimer, PT, INR, APTT, LSM, FIB‐4, DM, HTN and CHD cholesterol between the nonsignificant and significant fibrosis groups (Table 2). These variables were identified as potential predictors of significant liver fibrosis and were candidates for inclusion in the predictive model, warranting further detailed analysis and validation.

**TABLE 2:** Baseline characteristics and clinical parameters between patients with nonsignificant and significant liver fibrosis in the training cohort.

Variables	Nonsignificant fibrosis(S0/1) *n* = 206	Significant fibrosis (S≥2) *n* = 146	*p* Value
Age (year)	47 (39‐54)	53 (47.25‐60)	<0.001
BMI (kg/m^2^)	22.395 (20.298‐24.47)	22.805 (20.848‐24.06)	0.687
WBC (×10^9^/L)	4.15 (3.883‐4.588)	4.015 (3.692‐4.375)	0.009
RBC	126.5 (118.25‐137.75)	123 (113‐130)	0.002
HB (×10^9^/L)	38.15 (35.425‐41.075)	36.75 (34.225‐39.125)	0.003
Hct (%)	2.8 (2.082‐3.6)	2.6 (1.985‐3.35)	0.001
N (×10^9^/L)	1.74 (1.4‐2.195)	1.43 (1.143‐1.928)	0.314
LY (×10^9^/L)	209.922±62.765	174.774±72.151	<0.001
PLT (×10^9^/L)	5.168±1.666	4.537±1.807	<0.001
PAR	330.788 (217.012‐458.979)	280.75 (194.606‐432.683)	0.001
SII	0.01 (0.01‐0.02)	0.02 (0.01‐0.028)	0.112
NPR	114.915 (92.585‐145.688)	111.265 (82.905‐149.71)	0.001
PLR	1.615 (1.183‐2.08)	1.725 (1.282‐2.378)	0.434
NLR	14.4 (10.2‐21.05)	18.05 (12.5‐29.275)	0.061
TB (µmol/L)	4.95 (3.2‐8.5)	7.05 (4.6‐12.775)	<0.001
DB (µmol/L)	0.36 (0.31‐0.448)	0.41 (0.33‐0.538)	<0.001
DB/TB	49.5 (28‐86.5)	55.5 (30‐108.75)	0.002
ALT (U/L)	44 (26‐66)	61.5 (36‐120.25)	0.283
AST (U/L)	0.827 (0.636‐1.286)	1.138 (0.794‐1.513)	<0.001
AST/ALT	77.5 (34.25‐165.75)	135.5 (53‐376.75)	<0.001
GGT(U/L)	1.7 (0.81‐3.42)	1.71 (0.845‐4.547)	<0.001
GGT/AST	92 (66.25‐151)	127 (78‐209.25)	0.283
ALP(U/L)	40.85 (37.9‐44.3)	39.35 (36.2‐41.7)	0.001
ALB (g/L)	27.25 (23.925‐30.75)	28.7 (24.5‐33.7)	<0.001
GLB (g/L)	4.68 (4.025‐5.355)	4.37 (3.752‐5.22)	0.044
A/G	1.295 (0.97‐1.727)	1.22 (0.94‐1.76)	<0.001
TC (mmol/L)	1.275 (0.982‐1.57)	1.3 (0.912‐1.55)	0.043
TG (mmol/L)	2.73 (2.223‐3.178)	2.5 (1.968‐3.13)	0.435
HDL (mmol/L)	54.4 (9.57‐64.85)	56.3 (41.6‐70.25)	0.638
LDL (mmol/L)	4.715 (3.865‐8.125)	5.12 (3.795‐10.47)	0.039
Cr (µmol/L)	267 (211.325‐351.55)	272.7 (201.925‐336.675)	0.103
BUN (mmol/L)	190 (132‐223)	195.5 (136.5‐248.5)	0.634
UA (µmol/L)	88.5 (54.25‐110.428)	69 (50‐107.963)	0.515
LDH (U/L)	102.635 (67.992–113.375)	93.625 (59.432–108.38)	0.285
Creatine Kinase	4.05 (3.802–4.713)	4.045 (3.765–4.612)	0.106
eGFR (mL/min/1.73 m^2^)	139.5 (133.95–141.4)	139.4 (133.025–141.5)	0.085
K (mmol/L)	101.6 (93.875–103.475)	101.85 (96.625–103.8)	0.647
Na (mmol/L)	2.2 (1.875–2.31)	2.185 (2.012–2.28)	0.641
Cl (mmol/L)	0.89 (0.84–0.978)	0.88 (0.81–0.968)	0.528
Ca (mmol/L)	1.145 (0.982–1.52)	1.09 (0.932–1.372)	0.304
Mg (mmol/L)	23.35 (18.6–26.175)	23.65 (20.725–25.6)	0.150
P (mmol/L)	0.22 (0.22–0.33)	0.245 (0.22–0.55)	0.041
CO_2_ (mmol/L)	12.6 (12.2–13.3)	13.1 (12.4–13.8)	0.987
D‐dimer (mg/L FEU)	0.99 (0.92–1.067)	1.03 (0.952–1.13)	0.006
PT (s)	36.5 (34.4–39.8)	37.6 (35.15–40.875)	0.001
INR	2.73 (2.345–3.27)	2.625 (2.11–3.21)	<0.001
APTT (s)	18.6 (17.5–19.6)	18.65 (17.4–19.8)	0.018
FIB (g/L)	8.244 (6.088–10.719)	10.799 (8.195–17.661)	0.077
TT (s)	0.403 (0.239–0.661)	0.699 (0.414–1.297)	0.740
LSM (kPa)	1.53 (1.262–1.736)	1.387 (1.106–1.627)	<0.001
FIB‐4	22.49 (20.57–24.977)	22.43 (20.708–24.58)	<0.001
Gender (%)			0.452
Female	146 (70.87)	98 (67.12)	
Male	60 (29.13)	48 (32.88)	
DM (%)			<0.001
No	194 (94.17)	119 (81.51)	
Yes	12 (5.83)	27 (18.49)	
HTN (%)			0.014
No	180 (87.38)	113 (77.40)	
Yes	26 (12.62)	33 (22.60)	
CHD (%)			0.031
No	196 (95.15)	130 (89.04)	
Yes	10 (4.85)	16 (10.96)	
HLP (%)			0.388
No	121 (58.74)	79 (54.11)	
Yes	85 (41.26)	67 (45.89)	

Data are *n* (%) or percentage (95%).

Abbreviations: BMI, body mass index; WBC, white blood cell; RBC, red blood cell; HB, hemoglobin; Hct, hematocrit; N, neutrophil; LY, lymphocyte; PLT, platelet; PAR, platelet–albumin ratio; SII, systemic inflammation index; NPR, neutrophil‐to‐platelet ratio; PLR, platelet‐to‐lymphocyte ratio; NLR, neutrophil‐to‐lymphocyte ratio; TB, total bilirubin; DB, direct bilirubin; DB/TB, direct bilirubin/total bilirubin; ALT, alanine aminotransferase; AST, aspartate aminotransferase; AST/ALT, aspartate aminotransferase/alanine aminotransferase; GGT, γ‐glutamyl transferase; GGT/AST, γ‐glutamyl transferase/aspartate aminotransferase; ALP, alkaline phosphatase; ALB, albumin; GLB, globulin; A/G, albumin/globulin; TC, totalcholesterol; TG, triglyceride; HDL, high‐density lipoprotein; LDL, low‐density lipoprotein; Cr, creatinine; BUN, blood urea nitrogen; UA, uric acid; LDH, lactate dehydrogenase; eGFR, estimated glomerular filtration rate; PT, prothrombin time; INR, international normalized ratio; APTT, activated partial thromboplastin time; FIB, fibrinogen; TT, thrombin time; LSM, liver stiffness measurement; FIB‐4, fibrosis‐4 index; DM, diabetes mellitus; HTN, hypertension; CHD, coronary heart disease; HLP, hyperlipidemia.

### 2.2 Feature Extraction and Selection for Liver Fibrosis Prediction Based on Correlation Analysis, The Boruta Algorithm, and Least Absolute Shrinkage and Selection Operator Regression

To construct a robust and clinically interpretable model for predicting significant liver fibrosis, we employed a systematic feature selection pipeline that integrated conventional statistical techniques with advanced ML strategies.

First, we performed a Spearman correlation analysis to assess the associations between 56 candidate clinical variables and the severity of liver fibrosis. As illustrated in Figure 2A and Table S1, several variables were significantly correlated with fibrosis stage. Specifically, FIB‐4, LSM, age, AST/ALT, PT, INR, DB, AST, NPR, GGT, TB, DM, D‐dimer, APTT, DB/TB, HTN, ALP, GLB, and CHD were positively correlated with fibrosis severity, whereas ALB, A/G, PLT, LY, RBC, PAR, Hct, HB, WBC, Mg, FIB, LDL, P, NLR, eGFR, and TC were negatively correlated. These findings therefore suggest the feasibility of using these clinical variables as biomarkers for detecting liver fibrosis among patients with undiagnosed or etiologically unclassified CLD. Variables such as Ca, SII, LDH, Cr, gender, creatine kinase, TT, K, N, GGT/AST, PLR, TG, CO_2_, Cl, HLP, Na, ALT, UA, HDL, BUN, and body mass index (BMI) were not significantly correlated with fibrosis stage and were therefore excluded from further analyses.

**FIGURE 2:**
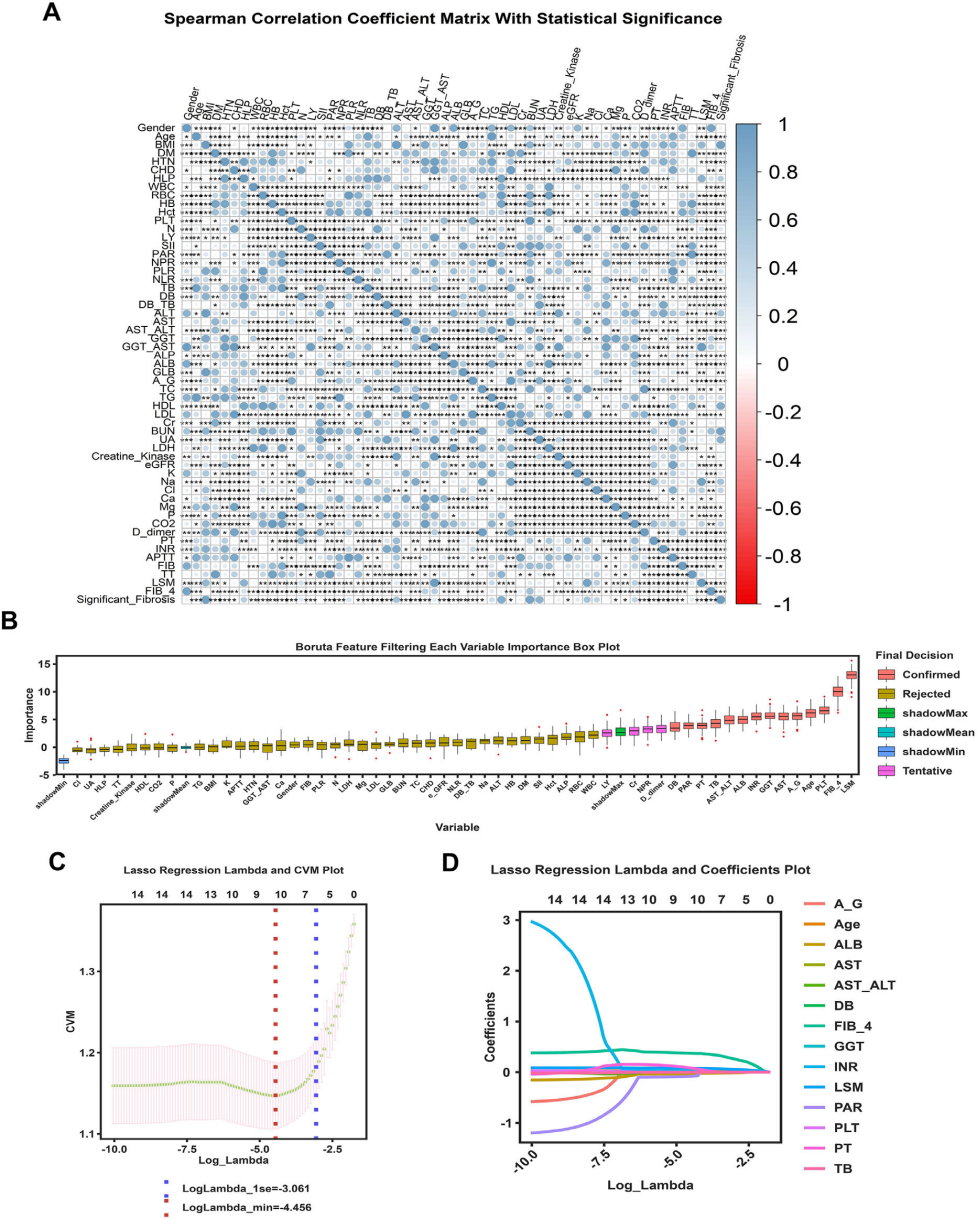
Feature selection for model construction. (A) Spearman correlation coefficient matrix of the 56 candidate clinical variables and significant liver fibrosis. The color and size of the circles indicate the direction and magnitude of the Spearman correlation coefficients, respectively, whereas asterisks denote the statistical significance of the corresponding correlations. The exact correlation coefficients and *p* values are provided in Table S1 (∗*p* < 0.05, ∗∗*p* < 0.01, and ∗∗∗*p* < 0.001). (B) Feature importance ranking using the Boruta algorithm. Orange bars indicate features retained as important, green bars represent rejected variables, and grey bars denote shadow features. Results are based on 1000 iterations. (C) LASSO regression CVM plot showing optimal lambda values selected via 10‐fold cross‐validation (lambda.min and lambda.1se). (D) Trace plot of LASSO regression coefficients as a function of log‐transformed lambda, identifying a sparse and nonredundant subset of predictors (age, PLT, AST, GGT, ALB, LSM, FIB‐4) used for model construction. Abbreviations: BMI, body mass index; WBC, white blood cell; RBC, red blood cell; HB, hemoglobin; Hct, hematocrit; N, neutrophil; LY, lymphocyte; PLT, platelet; PAR, platelet–albumin ratio; SII, systemic inflammation index; NPR, neutrophil‐to‐platelet ratio; PLR, platelet‐to‐lymphocyte Ratio; NLR, neutrophil‐to‐lymphocyte ratio; TB, total bilirubin; DB, direct bilirubin; DB/TB, direct bilirubin/total bilirubin; ALT, alanine aminotransferase; AST, aspartate aminotransferase; AST/ALT, aspartate aminotransferase/alanine aminotransferase; GGT, γ‐glutamyl transferase; GGT/AST, γ‐glutamyl transferase/aspartate aminotransferase; ALP, alkaline phosphatase; ALB, albumin; GLB, globulin; A/G, albumin/globulin; TC, total cholesterol; TG, triglyceride; HDL, high‐density lipoprotein; LDL, low‐density lipoprotein; Cr, creatinine; BUN; blood urea nitrogen, UA; uric acid, LDH; lactate dehydrogenase, eGFR; estimated glomerular filtration rate; PT, prothrombin time; INR, international normalized ratio; APTT, activated partial thromboplastin time; FIB, fibrinogen; TT, thrombin time; LSM, liver stiffness measurement; FIB‐4, fibrosis‐4 index; DM, diabetes mellitus; HTN, hypertension; CHD, coronary heart disease; HLP, hyperlipidemia.

To identify informative candidate predictors, following the correlation analysis, we proceeded with the next step in the feature selection process using the Boruta algorithm, a powerful Random Forest (RF)‐based method for feature selection. After performing 1000 iterations, Boruta confirmed the significance of 14 key variables (LSM, FIB‐4, PLT, age, A/G, AST, GGT, INR, ALB, AST/ALT, TB, PT, PAR, DB) on the basis of their importance scores significantly surpassing those of the shadow features (Figure 2B).

To further refine the feature set, reduce feature redundancy, and minimize the risk of overfitting, we applied Least Absolute Shrinkage and Selection Operator (LASSO) regression. LASSO uses regularization to minimize overfitting and enhances the model's predictive power by selecting a sparse set of features. By examining the cross‐validation error curve and the log lambda values (Figure 2C,D), we determined the optimal regularization strength and identified the most significant variables associated with liver fibrosis stage. This process allowed us to narrow the feature set down to the following key predictors: age, PLT, AST, GGT, ALB, LSM, and FIB‐4 (Table S2). Multicollinearity assessment of these predictors yielded VIF values ranging from 1.081 to 1.559. All the VIF values were less than 2, indicating that there was no evidence of problematic multicollinearity among the variables included in the final model (Table S3). These features were subsequently used to develop the liver fibrosis prediction models. This set of features serves as the foundation for the subsequent development of our liver fibrosis prediction model, which is both statistically robust and clinically relevant.

### 2.3 Comparative Evaluation of ML Algorithms for Accurate and Clinically Relevant Prediction of Significant Liver Fibrosis

To achieve accurate staging of liver fibrosis, we systematically evaluated 12 different ML algorithms to identify the most effective predictive model, and the detailed information on their hyperparameters is shown in Table S4. Comprehensive model performance metrics are presented in Table S5. On the basis of receiver operating characteristic (ROC) curve analysis, CatBoost demonstrated the best performance, yielding the highest area under the curve (AUC = 0.827), followed by LightGBM (AUC = 0.8087), RF (AUC = 0.7900), Logistic Regression (AUC = 0.7896), and XGB (AUC = 0.7881) (Figures 3A and S1 and Table S5). Notably, precision–recall (PR) curve analysis further confirmed the superior classification ability of CatBoost, with the highest PR area under the curve (PR‐AUC) of 0.804 (Figures 3B and S2). Beyond discrimination, CatBoost also showed the most favorable classification balance, with the highest F1‐score (0.7387) and Matthews correlation coefficient (MCC) (0.5889), together with a recall of 0.7069, specificity of 0.8710 and precision of 0.7736. These results indicate that CatBoost provided the most robust trade‐off between detecting patients with significant fibrosis and minimizing false‐positive classification (Table S5).

**FIGURE 3:**
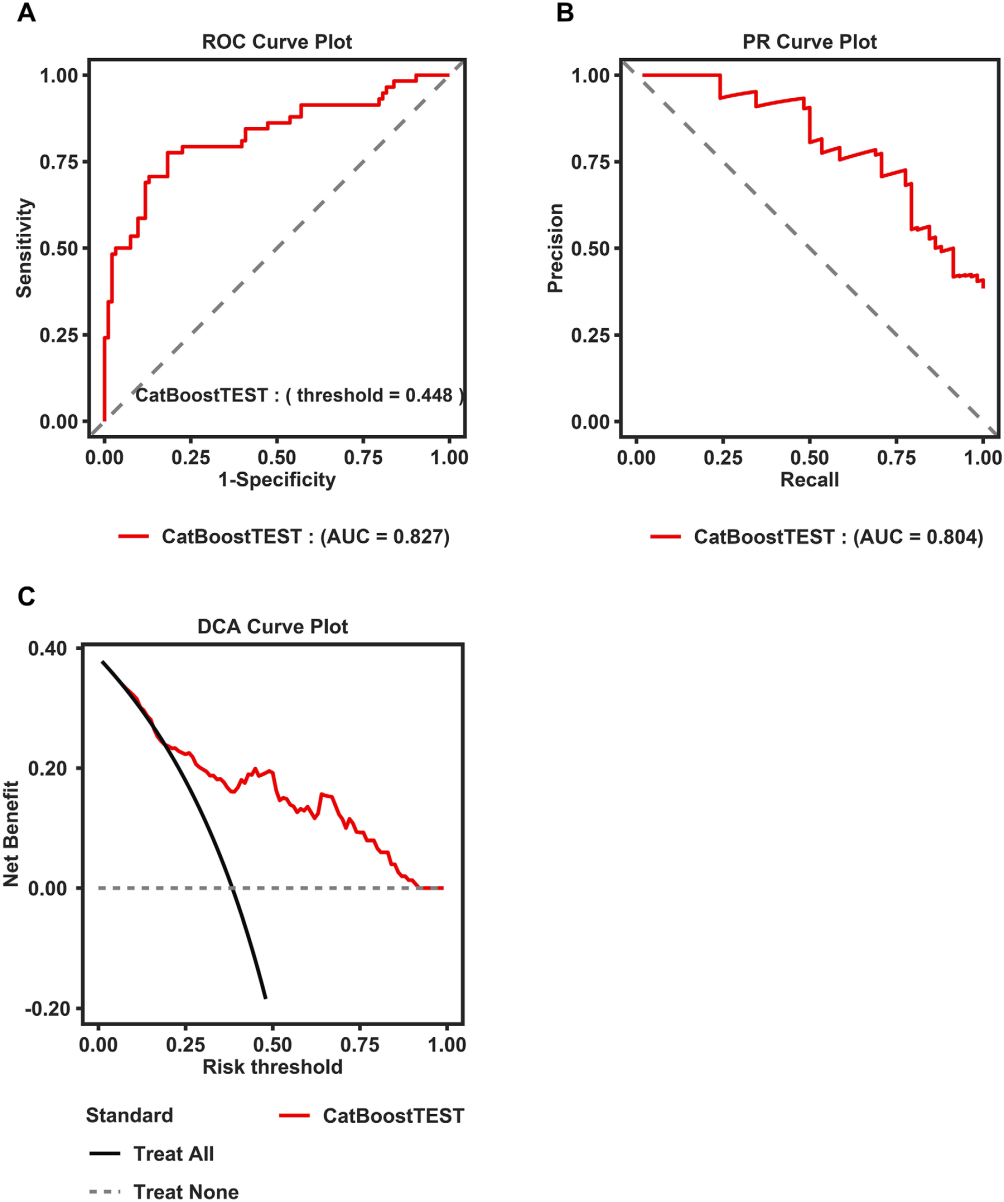
Performance evaluation of the CatBoost model in the independent test cohort. (A) Receiver operating characteristic (ROC) curve analysis of the selected CatBoost model for identifying significant liver fibrosis, showing an area under the ROC curve (AUROC) of 0.827. (B) Precision–recall (PR) curve analysis evaluating the model's performance in predicting patients with significant fibrosis, with a PR‐AUC of 0.804. (C) Decision curve analysis (DCA) demonstrating the clinical net benefit of the CatBoost model across different threshold probabilities, indicating potential clinical utility for noninvasive fibrosis risk stratification. Abbreviation: CatBoostTEST, Categorical Boosting Test.

In addition, decision curve analysis (DCA), a tool grounded in decision theory, was used to quantify the prospective clinical value of disease detection models, and revealed that CatBoost yielded the highest net clinical benefit across a wide range of threshold probabilities (Figures 3C and S3), highlighting its potential value in clinical decision‐making.

Collectively, these results provide strong evidence that CatBoost performs well in the clinical prediction of significant liver fibrosis, and it was used for all subsequent in‐depth analyses.

### 2.4 Fivefold Cross‐Validation Confirms Model Robustness

To assess the internal stability of the selected CatBoost classifier and to minimize the risk of information leakage, we performed fivefold cross‐validation under the same training‐only preprocessing and modeling framework, in which each iteration was trained using only the corresponding training folds and evaluated on the held‐out fold. Across the five held‐out folds, CatBoost maintained reproducible discriminatory performance, with Area Under the Receiver Operating Characteristic curves (AUROCs) of 0.8222, 0.8057, 0.7956, 0.7717, and 0.7692 corresponding to a mean AUROC of 0.7929 (Figure 4A and Table S6). The fold‐specific optimal thresholds ranged from 0.402 to 0.516, indicating that model performance was not dependent on a single data partition.

**FIGURE 4:**
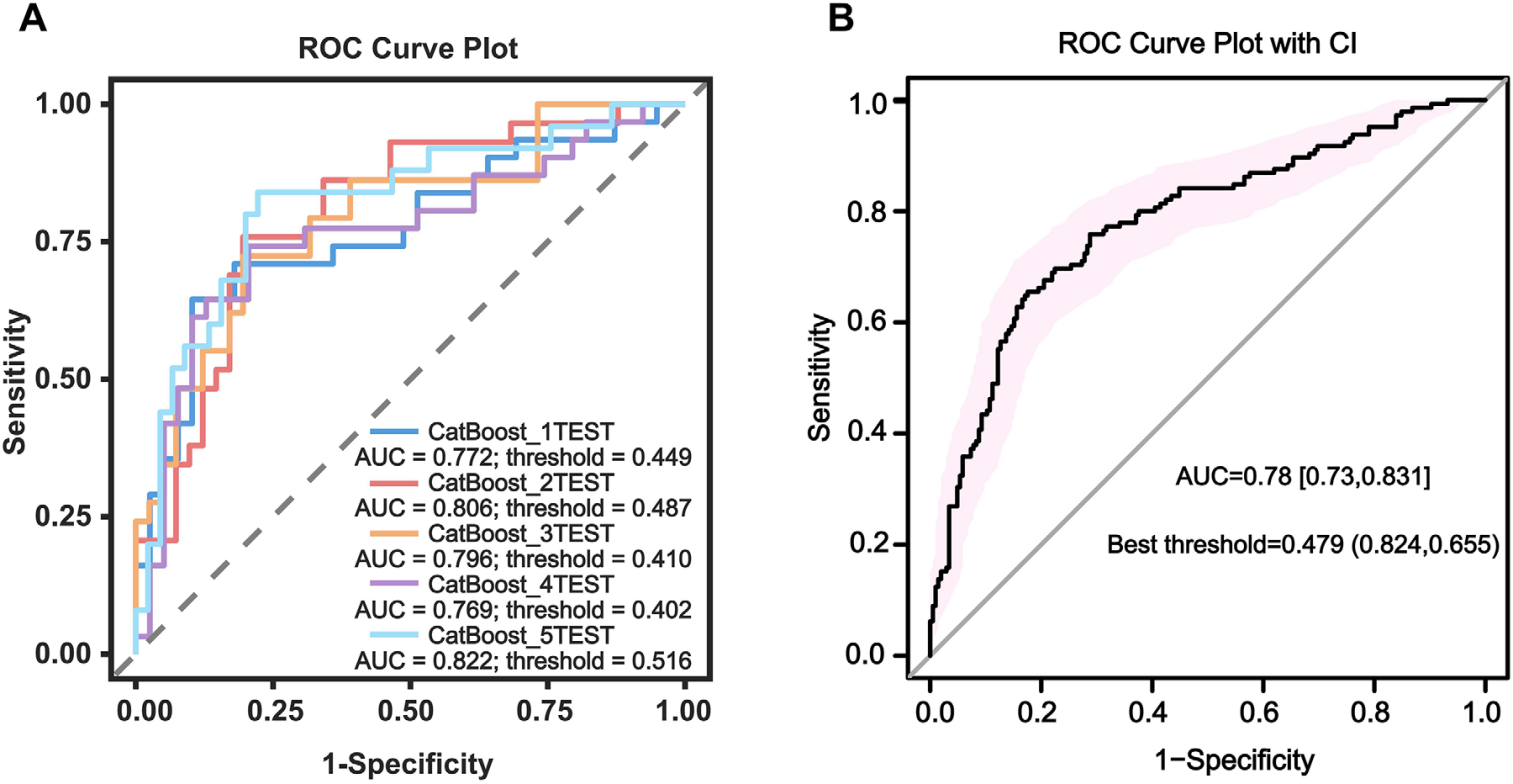
Internal validation using fivefold cross‐validation. (A) Fivefold cross‐validation ROC curves of the CatBoost model. Fold‐specific AUROCs ranged from 0.769 to 0.822; with fold‐specific optimal thresholds labeled. (B) Pooled ROC curve from aggregated cross‐validation predictions. The pooled AUROC was 0.78 (95% CI, 0.73–0.831); the optimal threshold of 0.479 yielded sensitivity of 0.824 and specificity of 0.655.

The operating characteristics were also broadly consistent across folds. The mean accuracy, recall, precision, specificity, F1‐score, and MCC were 0.7486, 0.6361, 0.7395, 0.8373, 0.6714, and 0.4895, respectively (Table S6). Notably, the model preserved relatively high specificity across the held‐out validation folds, supporting its ability to limit false‐positive classification while identifying individuals at risk of significant liver fibrosis.

In the pooled cross‐validation analysis, the aggregated ROC curve yielded an AUROC of 0.78 (95% CI, 0.73–0.831) (Figure 4B). At the optimal threshold of 0.479, CatBoost achieved a sensitivity of 0.824 and a specificity of 0.655 (Figure 4B), demonstrating a clinically meaningful balance between case detection and false‐positive control.

Together, these findings confirm the internal robustness of CatBoost and support its use as a stable noninvasive model for predicting significant liver fibrosis.

### 2.5 SHapley Additive exPlanations‐Based Interpretation Reveals Mechanistic Coherence Between Model Predictions and Fibrosis Pathophysiology

To clarify the decision logic of the CatBoost‐based liver fibrosis model, we applied SHapley Additive exPlanations (SHAP) analysis to quantify the contribution of each clinical variable to global model behavior and individual risk prediction. This approach enabled us to assess whether the model captured clinically meaningful fibrosis‐related signals rather than noninterpretable statistical patterns.

Globally, the SHAP importance plot revealed LSM and FIB‐4 as the leading predictors of significant liver fibrosis, followed by ALB, age, GGT, AST, and PLT (Figure 5A). The dominance of LSM and FIB‐4 score indicates that the model primarily relies on established noninvasive fibrosis surrogates, supporting the clinical plausibility of its predictive structure.

**FIGURE 5:**
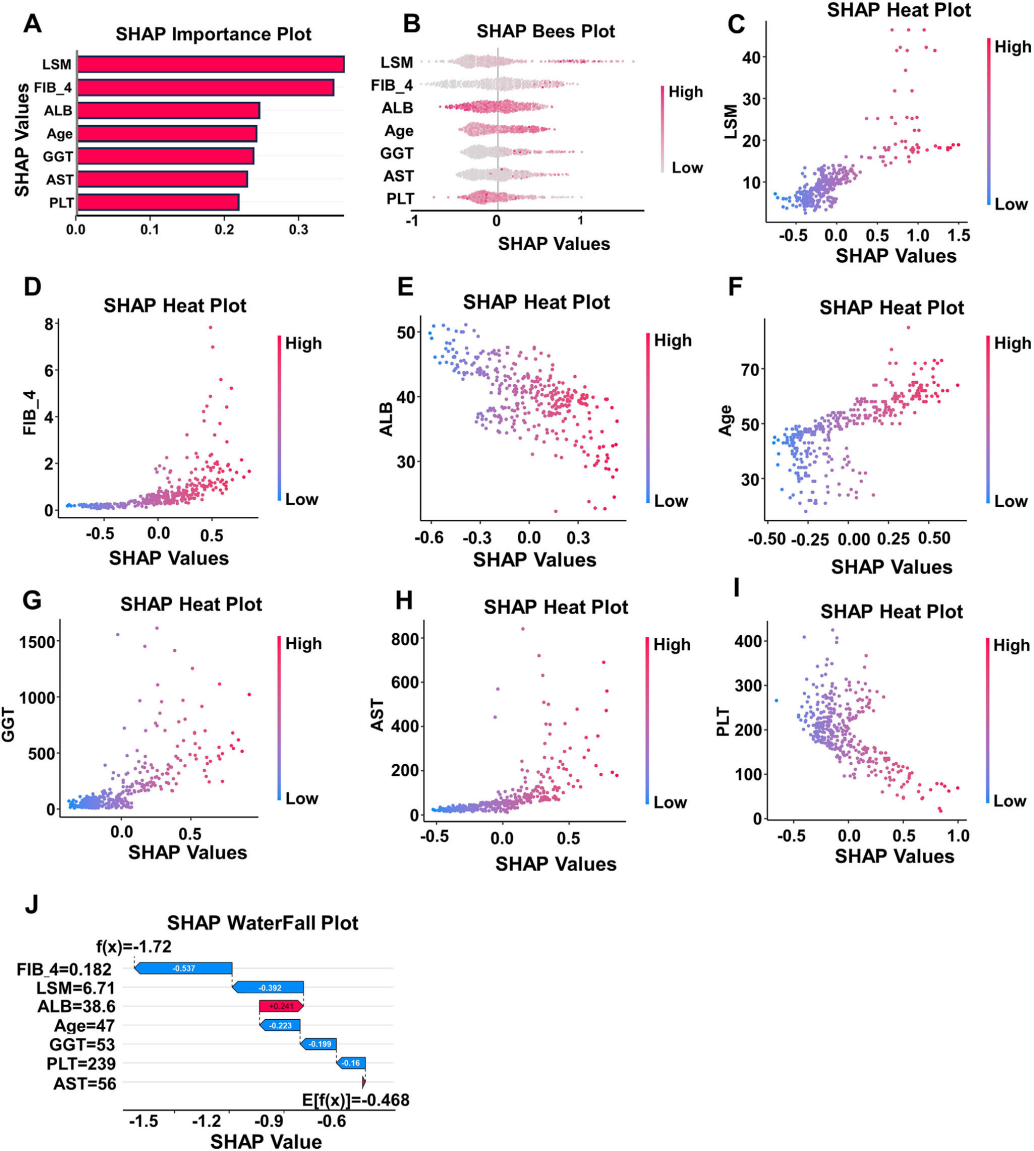
Model interpretability analysis using SHAP framework. (A) Global SHAP feature‐importance plot showing that LSM and FIB‐4 were the leading contributors to model prediction, followed by ALB, age, GGT, AST, and PLT. (B) SHAP beeswarm plot illustrating the direction and magnitude of feature effects across the cohort. Higher LSM, FIB‐4, age, GGT, and AST values generally increased the predicted probability of significant liver fibrosis, whereas higher PLT values reduced predicted risk. Lower ALB values contributed to higher fibrosis probability. (C–I) SHAP dependence plots showing feature‐specific effects for LSM, FIB‐4, ALB, age, GGT, AST, and PLT, respectively, highlighting nonlinear and clinically interpretable relationships between predictor values and model output. (J) SHAP waterfall plot for a representative patient, demonstrating how individual features shifted the prediction from the baseline output to the final patient‐specific fibrosis‐risk estimate. Low FIB‐4 and LSM, together with preserved PLT, decreased the predicted risk, whereas ALB and age provided opposing positive contributions, with GGT and AST adding liver injury‐related information. Abbreviations: LSM, liver stiffness measurement; PLT, platelet; GLB, globulin; PT, prothrombin time; INR, international normalized ratio; GGT, γ‐glutamyl transferase.

The SHAP beeswarm plot further defined the direction of these effects (Figure 5B). Higher LSM, FIB‐4, age, GGT, and AST values generally increased the predicted probability of significant fibrosis, whereas higher PLT values reduced the predicted risk. ALB showed an inverse pattern, with lower serum ALB levels contributing to a higher probability of significant fibrosis. These associations are consistent with key biological features of fibrosis progression, including increased liver stiffness, impaired hepatic synthetic function, hepatocellular or cholestatic injury, platelet depletion, and age‐related susceptibility.

The results of feature‐dependence analyses provided additional pathophysiological granularity. LSM and FIB‐4 showed positive relationships with SHAP values, indicating progressively greater risk contributions with increasing liver stiffness and fibrosis‐score burden (Figure 5C,D). In contrast, the serum ALB concentration was negatively associated with the predicted risk, with lower levels producing positive SHAP contributions (Figure 5E). Age displayed a gradual risk‐enhancing effect (Figure 5F), whereas GGT and AST showed dose‐dependent positive effects, suggesting that biochemical liver injury is linked to increased risk of fibrosis (Figure 5G,H). PLTs exhibited a strong inverse association, whereby lower platelet counts shifted predictions toward greater fibrosis probability, which is consistent with thrombocytopenia as a marker of advanced fibrotic remodeling and portal hypertension (Figure 5I).

At the individual level, the SHAP waterfall plot illustrated how the model generated a patient‐specific prediction (Figure 5J). In this representative case, low FIB‐4 and LSM values, together with preserved PLT, lowered the model output, whereas ALB and age provided opposing positive contributions. GGT and AST levels further provided case‐specific information related to liver injury. This individualized explanation shows that CatBoost integrates stiffness‐based assessment, composite fibrosis burden, hepatic synthetic function, biochemical injury markers, and hematologic status to estimate fibrosis risk.

Collectively, SHAP analysis demonstrated that the CatBoost model derived its predictions from variables that are biologically aligned with liver fibrosis progression. By capturing coherent axes of fibrosis biology—liver stiffness, fibrosis‐score burden, hepatic synthetic dysfunction, liver injury, platelet depletion, and age‐related vulnerability—the model achieved enhanced transparency, clinical credibility, and translational potential for the noninvasive identification of significant liver fibrosis.

### 2.6 Enhanced Discriminative Power of the ML‐Based Diagnostic Prediction Model Over Traditional Fibrosis Scores

To comprehensively assess the clinical performance of the newly developed ML‐based diagnostic prediction (DP) model, we conducted a head‐to‐head comparison against four conventional noninvasive fibrosis assessment tools—FIB‐4, the Forns Index, the GPR, and LSM—in the independent test cohort. The final DP model, which was developed in the training cohort, and all conventional scores were evaluated using the same histological endpoint of significant fibrosis (Scheuer stage S≥2). In the independent test cohort, the DP model showed the strongest overall discriminative performance, with an AUROC of 0.827 (95% CI, 0.754–0.900), outperforming LSM (0.745, 95% CI, 0.660–0.830), FIB‐4 (0.723, 95% CI, 0.636–0.810), the Forns Index (0.704, 95% CI, 0.615–0.794), and GPR (0.630, 95% CI, 0.537–0.723) (Figure 6 and Table S7). The upward shift of the DP model ROC curve indicates improved separation between patients with and without significant fibrosis compared with conventional single‐score approaches.

**FIGURE 6:**
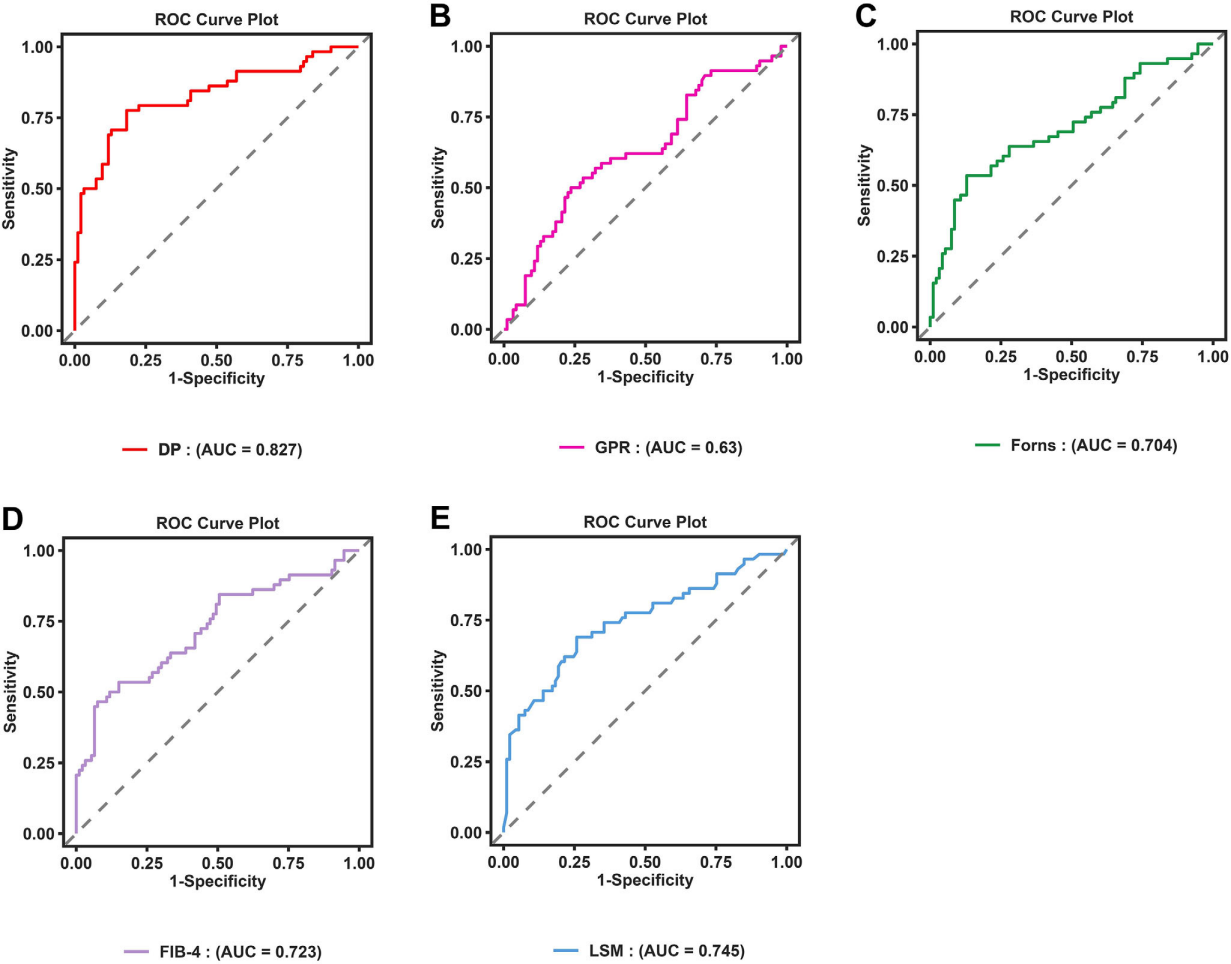
ROC curve analysis of the DP model and conventional noninvasive fibrosis assessment tools in the independent test cohort. (A) ROC curve of the CatBoost‐based DP model for identifying significant liver fibrosis, with an AUROC of 0.827. (B–E) ROC curves of conventional noninvasive fibrosis assessment tools, including GPR (B), Forns Index (C), FIB‐4 (D), and liver stiffness measurement (LSM) (E), with AUROCs of 0.630, 0.704, 0.723, and 0.745, respectively. Abbreviations: FIB‐4, fibrosis‐4 (age, ALT, AST, and platelet count); GPR, the GGT/platelet ratio; LSM, liver stiffness measurement; Forns, Forns Index.

Beyond the AUROC, the DP model also demonstrated a more balanced operating profile. It achieved the highest sensitivity (0.707), NPV (0.827), and accuracy (0.808) among all the evaluated models, while maintaining high specificity (0.871) and PPV (0.774). In contrast, FIB‐4 had the highest specificity (0.925) and PPV (0.794) but had substantially lower sensitivity (0.466), suggesting a greater risk of missed significant fibrosis. LSM achieved comparable sensitivity (0.690) but lower specificity (0.742) and accuracy (0.720), whereas GPR and the Forns Index showed weaker overall discrimination and classification balance (Table S7).

Overall, these findings support the use of the DP model as a precise and noninvasive tool that significantly outperforms previously established noninvasive prediction models. It holds strong potential for clinical translation by enabling individualized risk stratification and personalized prediction of liver fibrosis.

## 3 Discussion

This study asked a clinically direct question: can significant liver fibrosis be identified noninvasively in patients whose CLD has heterogeneous or ambiguous etiologies? To address this, using 503 biopsy‐confirmed cases (Figure 1), we developed a seven‐feature model incorporating age, PLT, AST, GGT, ALB, LSM, and FIB‐4, and benchmarked 12 machine‐learning algorithms. CatBoost showed the best overall performance and was selected as the final DP model, achieving an AUROC of 0.827 (95% CI, 0.754–0.900) in the independent test cohort (Figures 3 and S1–S4) and outperforming LSM, FIB‐4, Forns Index, and GPR (Figure 6 and Table S7).

In recent years, rapid advancements in artificial intelligence have driven significant progress in the application of ML algorithms for the diagnosis and staging of liver fibrosis. Despite these advancements, most current ML‐based fibrosis prediction models heavily rely upon imaging modalities, such as magnetic resonance elastography [18] or specialized serum biomarkers that are challenging to routinely measure in standard clinical practice, such as CXCL10 [19], hyaluronic acid [20], Type IV collagen [20, 21], and N‐glycomics [22]. Consequently, the necessity for sophisticated instrumentation, high assay costs, and limited medical resources restrict the widespread adoption and clinical implementation of these models, especially in primary care settings or resource‐constrained institutions. To overcome these limitations, we developed a novel noninvasive fibrosis prediction model that integrates routine serological parameters with cost‐effective LSM obtained via FibroTouch. Furthermore, previously reported ML models predominantly targeted distinct subsets of liver diseases with specific etiologies, including chronic hepatitis B^14^ or NAFLD [23, 24, 25]. However, in clinical practice, a substantial number of patients present with complex or atypical manifestations, making precise classification of their liver disease etiology challenging [26]. Our study specifically addresses this clinically important yet under‐investigated patient group classified as having “unclassified liver disease,” thus filling an important research gap by providing clinicians with an effective tool for the early screening of significant fibrosis in patients whose liver disease etiologies remain unclear. Consequently, our approach has considerable practical value.

The head‐to‐head comparison supports this clinical premise. The DP model achieved a higher AUROC (0.827) than LSM (0.745), FIB‐4 (0.723), Forns Index (0.704), and GPR (0.630) (Figure 6). It also achieved the highest sensitivity (0.707), NPV (0.827), and accuracy (0.808), while preserving high specificity (0.871) (Table S7). This balance is clinically relevant: FIB‐4 showed higher specificity but substantially lower sensitivity, whereas LSM alone showed comparable sensitivity but lower specificity and accuracy. Thus, the DP model appears to add value not by replacing established tests, but by integrating complementary stiffness‐based, biochemical, hepatic synthetic, and hematologic signals.

To further enhance clinical applicability and interpretability, our study employed a combined analytical approach integrating Spearman correlation analysis (Figure 2A and Table S1), the Boruta algorithm (Figure 2B), and LASSO regression for feature selection (Figure 2C,D). From these methods, we identified seven key predictive biomarkers (PLT, AST, GGT, ALB), plus age, LSM, and FIB‐4 (Figure 5A and Table S2), almost routinely assessed in clinical laboratories, which strongly align with established pathological mechanisms underlying liver fibrosis, For example, decreased platelet counts indicative of portal hypertension [27], and lowered albumin levels reflecting impaired hepatic synthetic capacity [28]. This selection process ensures that our model remains both streamlined and practically deployable in routine clinical scenarios.

Additionally, leveraging SHAP analysis [29] (Figure 5), we performed comprehensive global; relative contribution and directional impact of each feature (e.g., lower PLT, ALB and elevated AST, GGT, age, LSM, and FIB‐4 associated with increased fibrosis risk), thereby significantly enhancing clinical transparency and trust in the predictive outcomes. Notably, the development of our model strictly adhered to robust ML validation standards. Using data from 503 patients, we partitioned the dataset into training and testing sets in a 7:3 ratio. From a methodological perspective, the present dataset showed only moderate class imbalance, with significant fibrosis accounting for 40.56% of the overall cohort. We therefore preserved the original class structure and used stratified random splitting rather than applying synthetic resampling, while evaluating model performance with multiple complementary metrics to reduce class‐related assessment bias. Five‐fold cross‐validation further supported the internal robustness of the CatBoost model, showing a mean AUROC of 0.7929 across folds and a pooled AUROC of 0.78 (95% CI, 0.73–0.831), although the moderate recall (0.6361) indicates that further external validation and optimization are warranted (Table S6). Moreover, baseline characteristics were broadly comparable between the training and testing cohorts (Table 1), supporting the internal consistency of the modeling framework.

However, several limitations should be acknowledged. First, this was a single‐center retrospective study conducted at a tertiary hospital, and all 503 patients were enrolled from the same institution. Consequently, the study population may reflect center‐specific referral patterns, clinical workflows, laboratory testing conditions, and elastography practice, which may limit the external generalizability of the model. In addition, the cohort had an uneven etiological distribution, with autoimmune liver disease accounting for the largest subgroup, whereas viral, cholestatic, and hereditary metabolic liver diseases were less represented. Female patients also predominated. These characteristics may have influenced the feature distribution and decision boundaries learned by the model, and therefore the current findings should be interpreted cautiously when extrapolated to populations with different etiological spectra, demographic structures, disease severity distributions, or institutional practice settings. Second, although the CatBoost model showed favorable discrimination in the independent test cohort and in five‐fold internal cross‐validation, validation remained entirely internal. Accordingly, the robustness and transportability of the model across different hospitals, geographic regions, laboratory platforms, and LSM settings remain to be established. Prospective multicenter external validation and, if necessary, model recalibration will be essential before routine clinical deployment. Third, because this study was based on cross‐sectional data collected around the time of liver biopsy, the model was designed for fibrosis staging rather than for longitudinal monitoring of fibrosis progression, regression, or treatment response. Finally, although CatBoost showed the best discrimination and favorable decision‐curve performance, the calibration curve demonstrated visible deviation between predicted and observed risk (Figure S4). Therefore, the current model is better interpreted as a risk‐ranking and screening‐support tool than as a fully calibrated absolute‐risk calculator. Further external validation, recalibration, implementation studies, and prospective clinical impact assessment will be required before broad clinical adoption.

In conclusion, this study explicitly answers a clinically relevant question: in patients with CLD of uncertain or mixed etiology, a compact and interpretable CatBoost‐based DP model can improve noninvasive identification of significant liver fibrosis beyond conventional fibrosis scores. Its current strength lies in risk stratification and decision support, whereas its calibration limitations define a clear path for external validation, probability recalibration, and prospective clinical impact assessment.

## 4 Materials and Methods

### 4.1 Study Participants

A total of 503 patients with CLD who underwent ultrasound‐guided percutaneous liver biopsy at Union Hospital, Tongji Medical College, Huazhong University of Science and Technology between January 2017 and February 2024 were retrospectively enrolled. Inclusion criteria were as follows: (1) diagnosis of liver fibrosis consistent with the Chinese Guidelines for the Diagnosis and Treatment of Liver Fibrosis [30], (2) availability of histopathological staging based on ultrasound‐guided liver biopsy, (3) availability of the prespecified serological variables collected within ±7 days of the biopsy procedure and documented written informed consent for liver biopsy, and (4) complete and traceable clinical records. Exclusion criteria included: (1) substantial or untraceable missingness in key clinical or laboratory variables that precluded reliable analysis, (2) age under 18 years, (3) concurrent primary hepatic or extrahepatic malignancies, (4) coexisting severe comorbidities involving the cardiovascular, pulmonary, or renal systems, and (5) inadequate liver tissue for fibrosis staging based on histopathological evaluation.

### 4.2 Data Collection and Preprocessing

Baseline clinical data were systematically acquired at the time of enrollment for all eligible participants. Demographic characteristics—including age, sex, and BMI—were recorded upon hospital admission. Laboratory evaluations encompassed a broad panel of hematologic, hepatic, nephritic, and electrolyte‐related indicators, lipid metabolic biomarkers. Fasting peripheral venous blood samples were collected on the morning following admission using vacuum‐sealed tubes containing sodium citrate as an anticoagulant. All laboratory and serological tests were conducted within a 1‐week window before or after the liver biopsy procedure to ensure temporal alignment with histopathological assessment. Specimens were promptly transported to the hospital's central clinical laboratory, where they were processed and analyzed by certified laboratory personnel using standardized protocols and fully automated biochemical analyzers.

Prior to model development, data quality was systematically assessed. Patients with substantial or untraceable missingness in key clinical or laboratory variables were excluded according to the predefined eligibility criteria. For sporadic missing values in individual variables among the retained participants, mean imputation was performed. In accordance with the training‐only preprocessing framework, the mean values were estimated exclusively from the training cohort and subsequently applied unchanged to the independent test cohort to avoid information leakage. Potential outliers were verified against the original clinical records to identify possible transcription or data‐entry errors. Values confirmed to have been accurately recorded were retained because extreme laboratory or clinical measurements may represent genuine and clinically meaningful abnormalities in patients with CLD. Therefore, no observations were excluded solely on the basis of statistical outlier criteria.

Noninvasive tests were calculated as follows: FIB‐4 = (age(years) × AST (U/L))/((PLT count (10^9^/L) × (ALT (U/L))^1/2^) [31], Forns Index = 7.811−3.131 × ln(PLT count)(10^9^/L) + 0.781 × ln(GGT)(U/L) + 3.467 × ln(age)(years) − 0.014 × total cholesterol(mg/dL) [32], GPR = GGT/ULN of GGT/PLT(10^9^/L) × 100 [33].

### 4.3 Calculation of Sample Size

The sample size calculation was calculated using PASS software (Version 25.0.2), which was determined based on the following assumptions: Previous studies have reported that the proportion of patients with significant liver fibrosis accounts for approximately 17–30% of all fibrosis cases [34]. In the present study, we assumed a prevalence of 25% for significant fibrosis. Based on prior diagnostic models for liver fibrosis, the sensitivity and specificity were estimated at approximately 0.80 (null hypothesis, H_0_). The anticipated performance of our model was set at a sensitivity of 0.90 and a specificity of 0.85 (alternative hypothesis, H_1_), using a one‐sided test (H_1_ > H_0_) with a statistical power of 0.85 and a Type I error rate of 0.05. According to these parameters, the required total sample size was calculated to be 376, including 94 cases with significant fibrosis and 282 cases without significant fibrosis. Ultimately, this study enrolled 503 participants, comprising 204 in the significant fibrosis group and 299 in the nonsignificant group, thereby exceeding the calculated sample size requirement.

### 4.4 Pathological Diagnosis of Hepatic Fibrosis

Percutaneous liver biopsy was performed under real‐time ultrasound guidance after the patient had provided written informed consent. Eligible biopsy specimens measured >1.0 cm in length and contained at least five intact portal tracts. All samples were immediately fixed in 10% neutral‐buffered formalin, embedded in paraffin, and sectioned for histopathological evaluation. Reticulin staining and Masson's trichrome staining were performed following standard protocols. Histological staging of liver fibrosis was independently assessed by experienced hepatopathologists according to the internationally recognized Scheuer scoring system [35], which classifies fibrosis into five stages (S0–S4). Liver fibrosis stages S0 to S1 were classified as nonsignificant fibrosis, while stages S2–S4 were defined as indicative of clinically significant hepatic fibrosis [35].

### 4.5 ML Algorithms

After feature selection within the training cohort, 12 supervised ML algorithms were developed to predict significant hepatic fibrosis (Scheuer stage S≥2), including k‐nearest neighbors, multilayer perceptron, support vector machine, XGBoost, CatBoost, Naive Bayes, RF, LightGBM, Adaptive Boosting, Gradient Boosting Decision Tree, Decision Tree, and Logistic Regression. All models were implemented in R, and the hyperparameter settings used for each algorithm are summarized in Table S4.

The outcome distribution showed a moderate rather than severe class imbalance, with significant fibrosis accounting for 204 out of 503 patients (40.56%) overall, including 146 out of 352 (41.48%) in the training cohort and 58 out of 151 (38.41%) in the test cohort. Because the minority class was not markedly under‐represented and artificial resampling could distort the original clinicopathological structure of this biopsy‐confirmed cohort, no SMOTE‐ENN or other SMOTE‐type oversampling or undersampling procedure was applied. Instead, the full cohort was first divided into training and test cohorts by stratified random sampling in a 7:3 ratio to preserve the class distribution and to ensure that the independent test set remained untouched during model development. All preprocessing parameters, including the mean values used for missing‐data imputation, were estimated exclusively from the training cohort and then applied unchanged to the independent test cohort. Model performance was evaluated using multiple complementary metrics, including AUROC, PR‐AUC, specificity, precision, F1‐score, and MCC. The algorithm with the most favorable overall performance in the training/testing evaluation was selected as the final DP model.

To assess internal robustness and reduce the risk of information leakage, five‐fold cross‐validation was performed under the same training‐only preprocessing and modeling framework. In each iteration, model fitting was conducted using only the corresponding training folds, whereas the held‐out fold was used exclusively for validation. No validation fold or independent test‐set information was used in model training. To complement discrimination‐based assessment, the calibration of the final model was further examined in the independent test cohort, and clinical utility was assessed using DCA. Last, the SHAP method was used to assess the relative significance of predictor variables for enhancing the comprehensibility of our models [29].

For fair head‐to‐head comparison, the final DP model, after being developed in the training cohort, and the conventional noninvasive fibrosis assessment tools, including FIB‐4, Forns Index, GPR, and LSM, were all evaluated in the same independent test cohort using the same histological endpoint of significant fibrosis (Scheuer stage S≥2).

### 4.6 Statistical Analysis

Statistical analyses were conducted using SPSS software (version 28.0) and R software (version 4.0.3). Continuous variables were tested for normality and expressed as mean ± SD or median (interquartile range), and compared between groups using Student's *t*‐test or nonparametric tests, as appropriate. Categorical variables were expressed as counts and percentages [*n* (%)] and compared using the chi‐squared (*χ* ^2^) test. A two‐sided *p* value < 0.05 was considered statistically significant.

## Author Contributions

Ye Yang drafted the manuscript and performed the analysis. Yirui Hu and Ting Wei critically revised the manuscript and contributed substantially to the revision process, including responding to reviewer comments and refining the scientific content. Wenkang Gao collected the data and reviewed the statistical methods. Yue Chen, Yupin Tan, Yuanqing Zhu, Yonggang Yuan, Zhuang Cao, Yunfei Zhao, Zexuan Li, Jiake Che, Yue Yang, Xinyang Wang, Shengqi Yan, Weiyan Huang, Shuyan Wang, Fuyu Yang, Lijia Yin, Xiaotong Du, and Jiahao Liu collected clinical data. Rong Lin, Ling Yang, and Huikuan Chu conceptualized the study and critically revised the draft. All authors approved the final manuscript.

## Funding

This work was supported by grants from National Key R&D Program of China (No. 2022YFA1305600 to HC, No. 2023YFC2413804 to LY); National Natural Science Foundation of China under (No. 82470584 and No. 82000561 to HC, No. 82270614, No. 81974078 and No. 81570530 to LY); Science foundation of union hospital (No. 2021xhyn005) to HC.

## Ethics Statement

This research protocol complied with the Declaration of Helsinki and was approved by the Ethics Committee of Union Hospital, Tongji Medical College, Huazhong University of Science and Technology (UHCT21333).

## Conflicts of Interest

The authors declare no conflicts of interest.

## Supporting information




**Supporting File 1**: mco270990‐sup‐0001‐SuppMat.docx.

**Supporting File 2**: mco270990‐sup‐0002‐TableS1.xlsx.

## Data Availability

The data used to support the findings of this paper are available from the corresponding author upon reasonable request.
