# Supplementary material for: Machine Learning‐Driven Noninvasive Prediction of Liver Fibrosis in Unclassified Liver Disease
Source: MedComm (2020). 2026 Oct 4;7(10):e70990. doi: 10.1002/mco2.70990 (PMC13634176; doi:10.1002/mco2.70990)
Supplement: Supplementary file 1 [file MCO2-7-e70990-s001.docx]

**Machine learning-driven noninvasive prediction of liver fibrosis in unclassified liver disease**

Ye Yang^1^ , Yirui Hu^1^, Ting Wei^1^, Yue Chen^1^, Wenkang Gao^2,3^, Yupin Tan^1^, Yuanqing Zhu^1^, Yonggang Yuan^1^, Zhuang Cao^1^,Yunfei Zhao^1^, Zexuan Li^1^, Jiake Che^1^, Yue Yang^1^, Xinyang Wang^1^,Shengqi Yan^1^, Weiyan Huang^1^, Shuyan Wang^1^, Fuyu Yang^1^, Lijia Yin^1^, Xiaotong Du^1^, Jiahao Liu^1^, Rong Lin^1^, Ling Yang^1^, and Huikuan Chu^1^

^1^ Division of Gastroenterology, Union Hospital, Tongji Medical College, Huazhong University of Science and Technology, 1277 Jiefang Avenue, Wuhan, 430022, Hubei, China

^2^ Department of Critical Care Medicine, Zhongnan Hospital of Wuhan University, Wuhan, China.

^3^ Clinical Research Center of Hubei Critical Care Medicine, Wuhan, China

Ye Yang, Yirui Hu, and Ting Wei contributed equally to this works.

**Correspondence: Rong Lin** ([whuhlinrong@163.com](mailto:whuhlinrong@163.com)) | **Ling Yang** ([hepayang@163.com](mailto:hepayang@163.com); [yanglinguh@hust.edu.cn](mailto:yanglinguh@hust.edu.cn)) **| Huikuan Chu** (2012XH0827@hust.edu.cn)

**Supplementary Tables and Figures**

**Table S1. Spearman correlation coefficients and corresponding P values for associations between 56 candidate clinical variables and significant liver fibrosis.**

(A) **Matrix correlation analysis correlation matrix plot.**
(B) **Matrix Correlation analysis P value matrix plot.**

Abbreviations: BMI, body mass index, WBC, white blood cell, RBC, red blood cell, HB, hemoglobin, Hct, hematocrit, N, neutrophil, LY, lymphocyte, PLT, platelet, PAR, platelet-albumin ratio, SII, systemic inflammation index, NPR, neutrophil-to-platelet ratio, PLR, platelet-to-lymphocyte Ratio, NLR, neutrophil-to-lymphocyte ratio, TB, total bilirubin, DB, direct bilirubin, DB/TB, direct Bilirubin / total Bilirubin, ALT, alanine aminotransferase, AST, aspartate aminotransferase, AST/ALT, aspartate aminotransferase / alanine aminotransferase, GGT, γ-glutamyl transferase, GGT/AST, γ-glutamyl transferase / aspartate aminotransferase, ALP, alkaline phosphatase, ALB, albumin, GLB, globulin, A/G, albumin / globulin, TC, totalcholesterol, TG, triglyceride, HDL, high-Density Lipoprotein, LDL, low-Density Lipoprotein, Cr, creatinine, BUN, blood urea nitrogen, UA, uric acid, LDH, lactate dehydrogenase, eGFR, estimated glomerular filtration rate, PT, prothrombin time, INR, international normalized ratio, APTT, activated partial thromboplastin time, FIB, fibrinogen, TT, thrombin time, LSM, liver stiffness measurement, FIB-4, fibrosis-4 index, DM, diabetes mellitus, HTN, hypertension, CHD, coronary heart disease, HLP, hyperlipidemia, NAFLD, nonalcoholic fatty liver disease.

**Table S2. LASSO coefficients of candidate variables at the selected λ value.**

| **Variable** | **Coefficient at selected λ** | **Retained by LASSO** |
| --- | --- | --- |
| Age | 0.0144757160198043 | Yes |
| PLT | -0.000857347906487823 | Yes |
| PAR | 0 | No |
| TB | 0 | No |
| DB | 0 | No |
| AST | 0.000443287264930335 | Yes |
| AST/ALT | 0 | No |
| GGT | 0.000304358057658354 | Yes |
| ALB | -0.0134392568724147 | Yes |
| A/G | 0 | No |
| PT | 0 | No |
| INR | 0 | No |
| LSM | 0.0568397954346747 | Yes |
| FIB-4 | 0.268828309845334 | Yes |

Abbreviations: PLT, platelet count; PAR, platelet-albumin ratio; TB, total bilirubin; DB, direct bilirubin; AST, aspartate aminotransferase; GGT, γ-glutamyl transferase; ALB, albumin; A/G, albumin/globulin ratio; PT, prothrombin time; INR, international normalized ratio; LSM, liver stiffness measurement; FIB-4, fibrosis-4 index.

Note: Variables with coefficients shrunk to zero were not retained by LASSO. Variables with non-zero coefficients were selected into the final feature set.

**Table S3. Multicollinearity assessment of the predictors included in the final prediction model**

| Variable | VIF |
| --- | --- |
| Age | 1.165 |
| PLT | 1.464 |
| AST | 1.109 |
| GGT | 1.087 |
| ALB | 1.129 |
| LSM | 1.081 |
| FIB-4 | 1.559 |

Abbreviations: VIF, variance inflation factor; PLT, platelet count; AST, aspartate aminotransferase; GGT, γ-glutamyl transferase; ALB, albumin; LSM, liver stiffness measurement; FIB-4, fibrosis-4 index.

**Table S4. Details of hyperparameter for ML models.**

| **Models** | **Hyperparameter** |
| --- | --- |
| KNNCTEST | K= 5. |
| MLPTEST | hidden_layer_sizes=(100, 100, 100); activation="relu"; solver="adam"; alpha=0.0001; learning_rate_init=0.001; max_iter=200. |
| SVMTEST | kernel="rbf"; gamma=scale; C=1. |
| CatBoostTEST | eta=0.1; max_depth=10; eval_metric=logloss; max_iter=100. |
| NBTSET | estimator="multinomialNB"; alpha=1. |
| RFTEST | splitrule="gini"; n_trees=100; max_depth=3; min_samples_leaf=1. |
| Logistic TEST | penalty=none; solver="lbfgs"; max_iter=100; C=1. |
| AdaBoostTEST | base_estimator="DecisionTreeClassifier"; n_estimators=100; learning_rate=0.1; boosting_algorithm="SAMME.R". |
| LGBMTEST | eta=0.1, Weak learner type="gbdt". |
| XGBTEST | eta = 0.3, max_depth = 1, gamma =0, colsample_bytree= 0.6, min_child_weight= 1, subsample=0.5, nrounds=50 |
| GBDTEST | loss_type="log_loss"; n_estimators=100; learning_rate=0.1; subsample=1; criterion="friedman_mse"； min_samples_split=2; min_samples_leaf=1. |
| DesicionTreeTEST | splitrule="gini"; max_depth=3; min_samples_split=2; min_samples_leaf=1. |

Abbreviations: KNNCTEST, K-nearest neighbors Classification Test; MLPTEST, Multilayer Perceptron Test；SVMTEST，Support Vector Machine Test; CatBoostTEST, Categorical Boosting Test; NBTSET, Naive Bayes Test; RFTEST, Random Forest Test; AdaBoostTEST, Adaptive Boosting Test; GBDTEST, Gradient Boosting Decision Tree Test.

**Table S5. Performance comparison across twelve machine learning models.**

| **Model Name** | **AUROC** | **Recall** | **Specificity** | **Presicion** | **F1-Score** | **MCC** |
| --- | --- | --- | --- | --- | --- | --- |
| CatBoostTEST | 0.8270 | 0.7069 | 0.8710 | 0.7736 | 0.7387 | 0.5889 |
| LGBMTEST | 0.8087 | 0.6897 | 0.7634 | 0.6452 | 0.6667 | 0.4480 |
| RFTEST | 0.7900 | 0.6724 | 0.7527 | 0.6290 | 0.6500 | 0.4203 |
| LogisticTEST | 0.7896 | 0.6897 | 0.8065 | 0.6897 | 0.6897 | 0.4961 |
| XGBTEST | 0.7881 | 0.7241 | 0.7527 | 0.6462 | 0.6829 | 0.4684 |
| DesicionTreeTEST | 0.7336 | 0.4483 | 0.9355 | 0.8125 | 0.5778 | 0.4567 |
| MLPTEST | 0.7215 | 0.7759 | 0.5914 | 0.5422 | 0.6383 | 0.3590 |
| SVMTEST | 0.6965 | 0.6552 | 0.6022 | 0.5067 | 0.5714 | 0.2503 |
| KNNCTEST | 0.6748 | 0.6724 | 0.5806 | 0.5000 | 0.5735 | 0.2463 |
| NBTEST | 0.6528 | 0.4828 | 0.7204 | 0.5185 | 0.5000 | 0.2062 |
| AdaBoostTEST | 0.6513 | 0.6897 | 0.6129 | 0.5263 | 0.5970 | 0.2943 |
| GBDTTEST | 0.6415 | 0.5000 | 0.6882 | 0.5000 | 0.5000 | 0.1882 |

Abbreviations: CatBoostTEST, Categorical Boosting Test; LGBMTEST, Light Gradient Boosting Test; RFTEST, Random Forest Test; XGBTEST, Extreme Gradient Boosting Test; MLPTEST, Multilayer Perceptron Test; SVMTEST, Support Vector Machine Test; KNNCTEST, K-nearest neighbors Classification Test; NBTSET, Naive Bayes Test; AdaBoostTEST, Adaptive Boosting Test; GBDTEST, Gradient Boosting Decision Tree Test.

**Table S6. Detailed performance metrics of the CatBoost model in each fold of 5-fold cross-validation, including AUROC, accuracy, recall, precision, specificity, F1-score and MCC.**

| **Model Name** | **AUROC** | **Accuracy** | **Recall** | **Presicion** | **Specificity** | **F1-Score** | **MCC** |
| --- | --- | --- | --- | --- | --- | --- | --- |
| CatBoost_1TEST | 0.7717 | 0.7286 | 0.5161 | 0.8000 | 0.8974 | 0.6275 | 0.4547 |
| CatBoost_2TEST | 0.8057 | 0.7714 | 0.7241 | 0.7241 | 0.8049 | 0.7241 | 0.5290 |
| CatBoost_3TEST | 0.7956 | 0.7286 | 0.5517 | 0.7273 | 0.8537 | 0.6275 | 0.4302 |
| CatBoost_4TEST | 0.7692 | 0.7429 | 0.5484 | 0.8095 | 0.8974 | 0.6538 | 0.4832 |
| CatBoost_5TEST | 0.8222 | 0.7714 | 0.8400 | 0.6364 | 0.7333 | 0.7241 | 0.5503 |
| mean_scores | 0.7929 | 0.7486 | 0.6361 | 0.7395 | 0.8373 | 0.6714 | 0.4895 |

Abbreviations: CatBoostTEST, Categorical Boosting Test.

**Table S7. Comparative performance metrics of the DP model versus conventional noninvasive fibrosis scoring systems.**

| **Model Name** | **DP** | **FIB-4** | **GPR** | **LSM** | **Forns** |
| --- | --- | --- | --- | --- | --- |
| AUC  (95% CI) | 0.827  （0.754–0.900） | 0.723  (0.636 – 0.810) | 0.630  (0.537 – 0.723) | 0.745  (0.660 – 0.830) | 0.704  (0.615 – 0.794) |
| Sensitivity  (95% CI) | 0.707  （0.573–0.819） | 0.466  (0.397 – 0.914) | 0.500  (0.362 – 0.948) | 0.690  (0.431 – 0.828) | 0.534  (0.397 – 0.725) |
| Specificity  (95% CI) | 0.871  （0.785–0.932） | 0.925  (0.495 – 0.968) | 0.763  (0.290 – 0.892) | 0.742  (0.624 – 0.978) | 0.871  (0.699 – 0.946) |
| PPV  (95% CI) | 0.774  （0.638–0.877） | 0.794  (0.506 – 0.909) | 0.569  (0.445 – 0.706) | 0.625  (0.554 – 0.914) | 0.721  (0.573 – 0.850) |
| NPV  (95% CI) | 0.827  （0.737–0.896） | 0.735  (0.709 – 0.902) | 0.710  (0.679 – 0.903) | 0.793  (0.723 – 0.870) | 0.750  (0.702 – 0.825) |
| Accuracy | 0.808 | 0.750 | 0.660 | 0.720 | 0.740 |

**Abbreviations:** DP, diagnostic prediction model (CatBoostTEST-based); FIB-4, fibrosis-4 (age, ALT, AST and platelet count); GPR, the GGT/platelet ratio; LSM, liver stiffness measurement; Forns, Forns Index; AUC, area under the curve; CI, confidence interval; NPV, negative predictive value; PPV, positive predictive value.

**Figure S1. Receiver-operating characteristic curve (ROC) of twelve machine learning models.**

(A) Categorical Boosting Test (CatBoostTEST) vs Light Gradient Boosting Test (LGBMTEST); (B) CatBoostTEST vs Logistic TEST ; (C) CatBoostTEST vs Random Forest Test (RFTEST); (D) CatBoostTEST vs Extreme Gradient Boosting Test (XGBTEST); (E) CatBoostTEST vs DesicionTreeTEST; (F) CatBoostTEST vs Multilayer Perceptron Test (MLPTEST) ; (G) CatBoostTEST vs Support Vector Machine Test (SVMTEST); (H) CatBoostTEST vs K-nearest neighbors Classification Test (KNNCTEST); (I) CatBoostTEST vs Naive Bayes Test (NBTSET); (J) CatBoostTEST vs Adaptive Boosting Test (AdaBoostTEST); (K) CatBoostTEST vs Gradient Boosting Decision Tree Test (GBDTEST).

**Figure S2. Precision-recall (PR) curve of twelve machine learning models.**

(A) Categorical Boosting Test (CatBoostTEST) vs Light Gradient Boosting Test (LGBMTEST); (B) CatBoostTEST vs Logistic TEST ; (C) CatBoostTEST vs Random Forest Test (RFTEST); (D) CatBoostTEST vs Extreme Gradient Boosting Test (XGBTEST); (E) CatBoostTEST vs DesicionTreeTEST; (F) CatBoostTEST vs Multilayer Perceptron Test (MLPTEST) ; (G) CatBoostTEST vs Support Vector Machine Test (SVMTEST); (H) CatBoostTEST vs K-nearest neighbors Classification Test (KNNCTEST); (I) CatBoostTEST vs Naive Bayes Test (NBTSET); (J) CatBoostTEST vs Adaptive Boosting Test (AdaBoostTEST); (K) CatBoostTEST vs Gradient Boosting Decision Tree Test (GBDTEST).

**Figure S3. Decision curve analysis (DCA) of twelve machine learning models.**

(A) Categorical Boosting Test (CatBoostTEST) vs Light Gradient Boosting Test (LGBMTEST); (B) CatBoostTEST vs Logistic TEST ; (C) CatBoostTEST vs Random Forest Test (RFTEST); (D) CatBoostTEST vs Extreme Gradient Boosting Test (XGBTEST); (E) CatBoostTEST vs DesicionTreeTEST; (F) CatBoostTEST vs Multilayer Perceptron Test (MLPTEST) ; (G) CatBoostTEST vs Support Vector Machine Test (SVMTEST); (H) CatBoostTEST vs K-nearest neighbors Classification Test (KNNCTEST); (I) CatBoostTEST vs Naive Bayes Test (NBTSET); (J) CatBoostTEST vs Adaptive Boosting Test (AdaBoostTEST); (K) CatBoostTEST vs Gradient Boosting Decision Tree Test (GBDTEST).

**Figure S4. Calibration curve of twelve machine learning models.**

(A) Categorical Boosting Test (CatBoostTEST) vs Light Gradient Boosting Test (LGBMTEST); (B) CatBoostTEST vs Logistic TEST ; (C) CatBoostTEST vs Random Forest Test (RFTEST); (D) CatBoostTEST vs Extreme Gradient Boosting Test (XGBTEST); (E) CatBoostTEST vs DesicionTreeTEST; (F) CatBoostTEST vs Multilayer Perceptron Test (MLPTEST) ; (G) CatBoostTEST vs Support Vector Machine Test (SVMTEST); (H) CatBoostTEST vs K-nearest neighbors Classification Test (KNNCTEST); (I) CatBoostTEST vs Naive Bayes Test (NBTSET); (J) CatBoostTEST vs Adaptive Boosting Test (AdaBoostTEST); (K) CatBoostTEST vs Gradient Boosting Decision Tree Test (GBDTEST).
